# Variation in Foot Strike Patterns during Running among Habitually Barefoot Populations

**DOI:** 10.1371/journal.pone.0052548

**Published:** 2013-01-09

**Authors:** Kevin G. Hatala, Heather L. Dingwall, Roshna E. Wunderlich, Brian G. Richmond

**Affiliations:** 1 Hominid Paleobiology Doctoral Program, The George Washington University, Washington, D. C., United States of America; 2 Center for the Advanced Study of Hominid Paleobiology, Department of Anthropology, The George Washington University, Washington, D. C., United States of America; 3 Department of Biology, James Madison University, Harrisonburg, Virginia, United States of America; 4 Human Origins Program, National Museum of Natural History, Smithsonian Institution, Washington, D. C., United States of America; Universidad Europea de Madrid, Spain

## Abstract

Endurance running may have a long evolutionary history in the hominin clade but it was not until very recently that humans ran wearing shoes. Research on modern habitually unshod runners has suggested that they utilize a different biomechanical strategy than runners who wear shoes, namely that barefoot runners typically use a forefoot strike in order to avoid generating the high impact forces that would be experienced if they were to strike the ground with their heels first. This finding suggests that our habitually unshod ancestors may have run in a similar way. However, this research was conducted on a single population and we know little about variation in running form among habitually barefoot people, including the effects of running speed, which has been shown to affect strike patterns in shod runners. Here, we present the results of our investigation into the selection of running foot strike patterns among another modern habitually unshod group, the Daasanach of northern Kenya. Data were collected from 38 consenting adults as they ran along a trackway with a plantar pressure pad placed midway along its length. Subjects ran at self-selected endurance running and sprinting speeds. Our data support the hypothesis that a forefoot strike reduces the magnitude of impact loading, but the majority of subjects instead used a rearfoot strike at endurance running speeds. Their percentages of midfoot and forefoot strikes increased significantly with speed. These results indicate that not all habitually barefoot people prefer running with a forefoot strike, and suggest that other factors such as running speed, training level, substrate mechanical properties, running distance, and running frequency, influence the selection of foot strike patterns.

## Introduction

Among mammals, humans are particularly adept at running for long distances. Certain human ancestors may have used their cursorial abilities for hunting (e.g., persistence hunting) in order to gain access to high-quality foods and reap a competitive advantage over other animals [1]. Bramble and Lieberman [2] argue that anatomical features associated with endurance running are present in early members of the genus *Homo*, suggesting that the ability to run long distances may have played an important role in human evolution over the past 2 million years.

Unlike many contemporary people, the earliest human runners almost certainly did not use footwear. The earliest footwear known from the archaeological record dates to about 8300 years ago and comes from the Midwestern United States [3]. Indirect anatomical evidence that may be biomechanically linked to the advent of footwear, specifically the gracilization of pedal phalanges, has been found in some human populations dating back to about 30,000 years ago [4]. No known archaeological or paleontological evidence has suggested that footwear was used by early members of the genus *Homo*, which first appear in the fossil record around 2 million years ago (Ma), or by early *Homo sapiens*, which first appear around 200 thousand years ago (ka). Fossil footprints at Ileret, Kenya provide direct evidence of hominins walking barefoot in at least three occurrences around 1.52 Ma [5], [6]. Other sets of fossil footprints were made more recently by barefoot early modern humans at Nahoon Point and Langebaan Lagoon, South Africa around 120 ka [7], at Engare Sero, Tanzania in the late Pleistocene [8], [9], and also at Willandra Lakes, Australia around 19–20 ka [10].

Some modern human populations still do not habitually wear shoes. Many sources of data [11]–[14] show that at least some of these modern unshod groups differ from habitually shod populations in their foot anatomy (e.g., more splayed toes) and foot function (e.g., more evenly-distributed plantar pressure). In light of these findings and the relatively recent advent of footwear in human evolutionary history, it is imperative that we develop a thorough understanding of foot anatomy and foot function in modern habitually unshod groups. Only with such an understanding can we develop informed hypotheses regarding the evolution of human gaits. Further, the need to understand the mechanics of habitually unshod running is heightened by recent enthusiasm for barefoot running and the continuing debates over its advantages and disadvantages for modern runners.

A series of recent studies have reported favorable findings regarding health and biomechanical benefits associated with barefoot running. One study that examined the running gait of habitually unshod runners demonstrated that forefoot strikes (FFS) and some midfoot strikes (MFS) do not generate the high impact peaks caused by a rearfoot strike (RFS) [15]. While cushioning in modern running shoes helps mitigate the effects of these forces and allows (or even encourages) people to land on their heels, the authors pointed out that barefoot runners who use a RFS may be at greater risk of injury or discomfort. Consequently, they argued that habitually unshod runners utilize a different biomechanical strategy than people who run in shoes – barefoot runners more often strike the ground with their forefoot or midfoot first, thereby reducing impact peaks, while shod runners tend to land on their heels. In support of Lieberman and colleagues' [15] hypothesis, additional studies have shown that long-distance runners who FFS may be less susceptible to repetitive stress injuries than their counterparts who use a RFS [16], and that certain running injuries may be linked to high impact peaks [17], [18]. Furthermore, running with a FFS incurs no additional metabolic cost compared to a HS [19], [20]. The potential to simultaneously reduce impact forces and injury risks, at no additional metabolic cost, suggest that the use of a FFS during endurance running may have been important for our unshod human ancestors.

However, some of the habitually unshod runners studied by Lieberman and colleagues [15] preferred a MFS or a RFS, highlighting the importance of considering other variables that may affect foot strike postures. For example, Nigg and colleagues [21] showed that when habitually shod runners increase speed, they alter the position of their foot at strike in order to cope with the higher collision forces associated with that greater speed. This hypothesis was supported by Keller and colleagues [22], who reported that habitually shod runners who used predominantly a RFS when running at speeds 5 m/s or slower, preferred a FFS at 6 m/s or faster. Together, these studies suggest that foot strike patterns are at least in part dependent upon running speed. The self-selected endurance running speeds of the habitually unshod runners studied by Lieberman and colleagues [15] happened to fall between 5 and 6 m/s, the same interval in which the runners studied by Keller and colleagues [22] altered their preferred patterns of foot strike. This leaves open the possibility that running foot strike patterns of habitually barefoot people are similarly influenced by running speed. In addition, the Kalenjin runners studied by Lieberman and colleagues [15] all ran more than 20 km per week on hard surfaces, and factors such as running distance and substrate mechanical properties also probably influence foot strike patterns.

We set out to investigate further foot strike patterns among a different group of habitually unshod people who run less often than the Kalenjin and to test for a relationship between foot strike patterns and running speed. Kinematic and plantar pressure data were collected from a group of 38 habitually unshod Daasanach adults from northern Kenya to test the hypothesis that habitually barefoot individuals tend to use a FFS, rather than a RFS, at their self-selected endurance running speeds. In light of previous research [21], [22] that associated changes in strike patterns with changes in running speed, we explored the alternate but not mutually exclusive hypothesis that strike patterns in habitually unshod runners are influenced by speed, with higher incidence of forefoot striking at higher speeds.

## Results

Data were collected from 38 Daasanach subjects (19 male, 19 female) who ran along a 15-meter trackway with a pedal pressure pad placed midway along its length. Each subject was asked to run along this trackway at least 3 times at their self-selected (comfortable) endurance running pace, and 3 more times at a faster pace. Strike pattern data were collected from all 38 subjects, while kinematic and kinetic data were collected from a subset of 18 (8 male, 10 female; see Materials and Methods). Running speeds ranged from 2.15–6.63 m/s (Froude numbers of 0.52–4.77).

**Figure 1:**
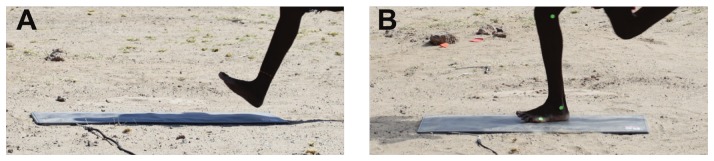
Close-up images of subjects using a rearfoot strike (A) and a midfoot strike (B). Most Daasanach subjects used a rearfoot strike (A) at their self-selected endurance running speeds, rather than a midfoot strike (B) or a forefoot strike (not shown).

**Figure 2:**
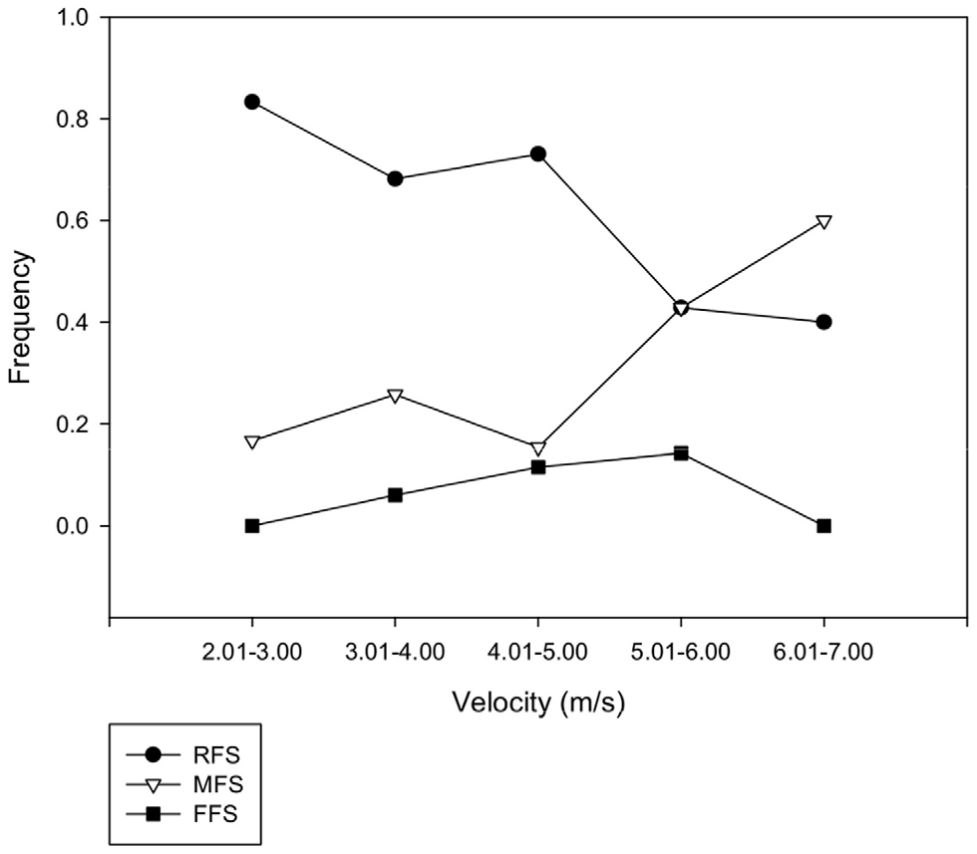
Frequencies of strike patterns by running speed. The majority of the Daasanach sample group used a RFS (black circles) when running at Froude speeds less than 2.5. For Froude speeds greater than 2.5, RFS and MFS (white circles) running were observed with equal frequencies. FFS running (black triangles) was most frequently observed at the highest speeds (Froude >3.5), but this pattern was never used by the majority of the Daasanach sample group.

When running at their endurance running speeds, the Daasanach subjects used a RFS in 96 of 133 trials (72%) and used a MFS in 32 of 133 trials (24%; Figure 1; Table 1). Subjects very rarely used a FFS at their self-selected running speeds (5 of 133, or 4%, of all trials). A further categorical breakdown of running speeds showed that the Daasanach used predominantly a RFS at velocities of 5.0 m/s and less. At speeds of 5.01–6.00 m/s, our sample group used a RFS and MFS with equal frequencies and at speeds between 6.01 and 7.00 m/s, the majority employed a MFS (Figure 2; Table 2). The incidence of a FFS was greatest at running speeds between 5.01 and 6.00 m/s (14% of trials) but this running style was never used by the majority of our subjects at any speed. A logistic regression analysis revealed that the influence of speed (velocity) on strike type was statistically significant (p = 0.0368). These results therefore indicate that not all habitually unshod individuals prefer to use a FFS when running at their self-selected running speeds. They show that our sample group consistently preferred a RFS or MFS over a FFS even when sprinting.

**Table 1:** Foot strike patterns of Daasanach (N = 38) over 133 trials at preferred endurance running speeds.

Strike Type (%)			
Rearfoot strike (RFS)	Midfoot strike (MFS)	Forefoot strike (FFS)	Average speed (m/s)
72	24	4	3.3±0.4*

* average based on data from 18 subjects; see Materials and Methods.

**Table 2:** Frequencies of foot strike patterns across running speeds.

Velocity (m/s)	Strike Type (% of trials)		
	Rearfoot strike (RFS)	Midfoot strike (MFS)	Forefoot strike (FFS)
2.01–3.00	83	17	0
3.01–4.00	68	26	6
4.01–5.00	73	15	12
5.01–6.00	43	43	14
6.01–7.00	40	60	0

**Figure 3:**
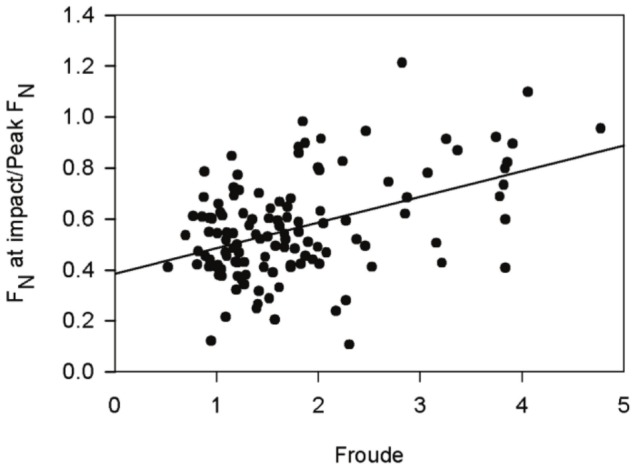
Least-squares regression of impact force by running speed. A significant relationship was found between the relative magnitude of impact forces and running speed (y = 0.38+0.10×; r^2^ = 0.20, p<0.0001).

However, our results do support the hypothesis that a FFS reduces the magnitude of impact forces relative to a RFS [15]. As predicted by previous analyses of running gait [21], [22], we found a significant but weak relationship between relative impact forces (calculated as normal force at strike divided by peak normal force) and speed (ordinary least-squares, r^2^ = 0.20, p<0.0001; Figure 3). Examining the residuals from this regression suggests that, on average, individuals using a FFS experienced lower relative impact forces than would be predicted by speed alone (Table 3). This was not the case for individuals using a RFS or MFS, who on average experienced equal and higher relative impact forces, respectively, than predicted. These results suggest that the adoption of a FFS, albeit rare in our sample group, reduced the impact forces experienced at foot strike.

**Table 3:** Mean residuals (and 95% confidence limits) from regression of relative impact force by running speed.

Strike type	N	Mean residual	Lower 95% CL	Upper 95% CL
RFS	83	0.00	−0.03	0.04
MFS	33	0.03	−0.04	0.10
FFS	8	−0.21	−0.39	−0.04

## Discussion

Our results indicate that not all habitually unshod people prefer a FFS or MFS at their preferred endurance running speeds. Rather, the Daasanach subjects in this study preferred a RFS at their self-selected endurance running speeds, and thus differed from the Kalenjin runners studied by Lieberman and colleagues [15]. It is intriguing to note that the Daasanach resembled habitually shod runners in that they showed a tendency to switch to a MFS or FFS at sprinting speeds [21], [22]. Several factors could explain the different foot strike patterns observed in these two habitually unshod Kenyan populations. For example, running speeds differed in the two studies. The Daasanach in our study selected endurance running speeds that averaged about 3.3 m/s (8:08 per mile), while the Kalenjin runners in theirs [15] averaged speeds of about 5.1–5.9 m/s (4:33–5:16 per mile). Among the Daasanach, although it was never the predominant pattern, we did observe a higher frequency of FFS running at speeds greater than their preferred endurance running paces. However, speed alone cannot explain these differences because recent research among Kalenjin runners indicates that they predominantly FFS at a wide range of speeds (2.4–6.0 m/s; 4:28–11:11 per mile), with no significant effects of running speed on strike type [DE Lieberman, personal communication].

Other factors also likely contribute to the higher incidence (91%) of FFS in the adult Kalenjin runners [15] compared with that observed (up to 14%; Table 2) when the Daasanach adults ran barefoot. For example, substrate compliance has been shown to influence foot strike patterns [23]. More compliant substrates would likely result in lower impact peaks; if the attenuation of impact forces plays an important role in the selection of foot strike patterns [15]–[18], then runners may make less adjustment to their strike patterns on more compliant substrates. If the Daasanach typically run on softer substrates than the Kalenjin (the Daasanach in our study ran on a firm natural surface), this may have led to their tendency towards heel-strike running. The level, frequency, and type of running may have also influenced the preferred strike patterns of the Kalenjin and Daasanach runners. The habitually barefoot Kalenjin studied by Lieberman and colleagues [15] run many kilometers on a daily basis and all ran a minimum of 20 km/week, but the Daasanach adults studied here do not run as frequently or as far as the Kalenjin. Training at a high intensity, running tens of kilometers each week, may elevate the risk of repetitive stress injuries such that it necessitates adjustments to one's running gait. For those who run less often and for shorter distances (e.g., the Daasanach compared to the Kalenjin), running may pose less of a threat to health and it may not be necessary, and never occur to those runners, to adopt a posture that reduces the impact peaks associated with RFS running. The influences of these factors on the preferred foot strike patterns of habitually unshod runners are currently unknown and warrant further investigation.

Testing hypotheses about the foot strike patterns used by early hominins is very difficult, as there are limitations in relating modern experimental work to the fossil record. The application of these experimental results to hypotheses about running techniques used through human evolution raises two questions. First, how far and how frequently did our ancestors run? The Kalenjin experimental subjects of Lieberman and colleagues [15] were runners, many training for competition, who may have necessarily adopted a FFS in order to avoid injuries associated with their frequent running over long distances [16]–[18]. The avoidance of high impact forces associated with running, especially on relatively firm substrates, may become more important among populations that regularly run for such long distances. Our Daasanach subjects certainly run less frequently than the Kalenjin, and perhaps habitually unshod people can afford to use a RFS at endurance running speeds if they do not run as part of their regular daily routine. Second, how fast did our ancestors run? Our results from the Daasanach, alongside the results of other research, suggest that the selection of particular foot strike postures may be dependent upon speed. If our ancestors' typical endurance running speeds were in the range of the Kalenjin studied by Lieberman and colleagues [15] (about 5.1–5.9 m/s or 4:33–5:16 per mile), then they may have relied upon a FFS. If they ran at slower speeds (the Daasanach ran at about 3.3 m/s or 8:08/mile), then RFS running may have been more frequent. However, because recent research has revealed that strike patterns among the Kalenjin are not dependent upon speed [DE Lieberman, personal communication], then it is possible that factors other than speed were more influential to the selection of running strike patterns by early hominins.

It is not clear which experimental sample, if either, represents a better ‘model’ for the distances and frequencies of running in early humans. It has been hypothesized that endurance running was important to our ancestors because it would have provided a means of acquiring high-quality foods (i.e., meat) through persistence hunting [1], [2]. Recent ethnographic research on persistence hunting by modern hunter-gatherers living in the central Kalahari documented successful persistence hunts ranging in distance from about 25–35 km [24], but it is unclear how often those hunts occur. Persistence hunts are not practiced by the Hadza hunter-gatherers in Tanzania [25]. However, the relevance of recent ethnographic data to these questions is limited because modern hunter-gatherers use relatively recent inventions (e.g., projectile weapons) and their diets and practices have been influenced by neighboring people who are not hunter-gatherers [26]. Regarding speed, the only data that exist for the Kalahari hunters are averaged over interspersed bouts of running and walking (1.67–2.78 m/s, or 9:39–16:15/mile) thereby making it difficult to draw biomechanical hypotheses from these data. Based on our results, if speed was a factor that influenced running foot strike patterns in early hominins, as it does in the Daasanach, and early hominins typically performed persistence hunts at speeds more similar to the preferred endurance running paces of the Daasanach, then this behavior may have been associated with predominantly RFS, rather than FFS running. Likewise, the opposite could be true if higher running speeds were maintained for persistence hunting. Predictions based on estimated costs of transport in humans and other mammals have suggested optimal pursuit speeds of 3.0 m/s for persistence hunting 10 kg prey, 4.2 m/s for 60 kg prey, and 5.3 m/s for prey over 200 kg [27]. More data is required on the types of prey that early hominins may have hunted using this strategy before any robust estimate can be made of the speeds used by the hominins that may have practiced this behavior. Bramble and Lieberman [2] also proposed the alternate hypothesis that endurance running was originally selected for its use in competitive scavenging. Higher running speeds may have been more important if they were following migrating ungulates [26] or if it was necessary to scavenge carcasses while simultaneously avoiding predators (but see [25], [26]). In such a scenario, higher-speed endurance running during scavenging may have led to a prevalence of FFS, rather than RFS running.

It is also unclear which experimental sample's environment might represent a better model for the substrates typical of those on which early humans ran. In a study of habitually shod runners, the frequency of rearfoot strike changed from 23.3% on asphalt to 54.3% on grass [23]. Although not as soft as grass, the natural surfaces on which the Daasanach and Kalenjin ran are certainly more compliant than the asphalt or concrete surfaces on which people typically run in heavily developed regions. However, data are not available on the compliance of substrates typical of ancient hominin environments.

Our results make it clear that more work with habitually unshod populations is needed to determine the extent to which variables such as running speed, running distance, substrate mechanical properties, and other factors might influence foot strike patterns during barefoot running. To date, our study and that of Lieberman and colleagues [15] are the only ones to examine running mechanics in habitually barefoot groups, and these studies' observations differ in important ways. It will be necessary to understand the extent and nature of the variation in running mechanics across unshod populations in different geographical areas, on different substrates, and with different behavioral repertoires. With a broader understanding of the factors that influence foot strike patterns in habitually unshod populations, we may be able to refine hypotheses regarding how running may have shaped the evolution of our anatomy, as well as the potential advantages and disadvantages of different strike patterns in modern runners.

## Materials and Methods

### Ethics Statement

The George Washington University's Institutional Review Board (IRB) granted approval for the human experimental study described in this paper, which was titled “Biomechanics of the human foot.” All subjects gave their written consent to participate, a procedure approved by the aforementioned IRB committee.

### Experimental Setup

We cleared an approximately 15-meter long trackway on an area of flat, open ground. The surface consisted of the firmly-packed sand that is typical of the savannah environment of the Ileret area. In the middle of this trackway, we placed a 1-meter long plantar pressure pad (RSScan International Footscan; recording frequency 252 Hz). We set up a video camera perpendicular to the trackway at a distance of 8 meters from the edge of the pressure pad. In 2010, we recorded video at 60 Hz using a Canon ZR50MC video camera. In 2011, we recorded high-speed video at 210 Hz using a Casio Exilim EX-FH20 camera. Because the slower-speed (60 Hz) videos from 2010 were at too low a rate to identify the precise moments of foot strike, kinematic analysis was restricted to the 2011 high-speed (210 Hz) video data (18 subjects; 8 male, 10 female). To calibrate the trackway for kinematic analyses, we placed two stakes 3 meters apart with the first at the beginning of the pressure pad and the second 2 meters beyond the pad.

### Subjects and Protocol

Over the course of two field seasons (2010 and 2011) we recruited 38 adults (19 male, 19 female) from the Daasanach tribe living in and around the town of Ileret, Kenya. We measured the height, weight, and functional leg length (greater trochanter height) of each subject. Each subject then passed over the trackway, including the pressure pad, for at least three trials at self-selected ‘endurance’ running speeds (they were asked to run at a comfortable pace at which they could run for a long distance). Then they conducted at least three more trials at self-selected sprinting speeds. These speeds were later quantified from high-speed digital video. Trials were discarded and repeated if subjects targeted the pressure pad or otherwise altered their gait in any way. All subjects missed the pressure pad in one or more trials, suggesting that they were not targeting the pressure pad.

### Analysis

The posture of the foot at strike was identified using data collected from the plantar pressure pad. We examined the first frame of data recorded, which represented the distribution of pressure the instant at which the foot struck the pad. A RFS was identified as a trial in which the first data frame included pressure on the heel only. A FFS was identified as a trial in which plantar pressure was exerted only on the forefoot (metatarsal heads) at touchdown. The heel almost always contacted the ground in these cases, but not until later in the stance phase. A MFS was considered to include trials where initial contact occurred at the lateral midfoot (cuboid/base of the 5^th^ metatarsal), and also those trials where initial contact included both the heel and forefoot. Measurements of peak normal force, over the duration of the stance phase, were extracted for each trial. We also identified the impact peak (see [15]) in each trial and extracted the instantaneous normal force at that moment. As in the study by Lieberman and colleagues [15], if we could not identify a distinct peak, then we extracted the normal force from the average percentage of stance phase at which impact peaks occurred (7%). The normal force at the impact peak was divided by peak normal force in order to generate a standardized measure of impact loading that could be compared across subjects. While this standardization deviates from the ‘typical’ procedure of dividing by body weight, it does represent a normalized relative magnitude of the impact peak because peak normal force over the entire duration of stance phase should not necessarily vary according to the type of foot strike [15].

To analyze kinematic data, high-speed videos collected during the 2011 field season were imported into ImageJ (http://rsbweb.nih.gov/ij/). Running speed was calculated by measuring the time it took a digitized marker on each subject's sternum to traverse the 3-meter long calibrated portion of the trackway. We converted these measures to dimensionless Froude numbers, using the formula provided by Alexander and Jayes [28], where Froude = v^2^/gh (v = velocity, g = gravitational acceleration, h = hip height). ImageJ was also used to measure stride length and stride frequency (the inverse of time elapsed during one stride) for each trial. All statistical analyses were conducted using JMP 9.0 statistical software.

## References

[1] Carrier DR (1984) The energetic paradox of human running and hominid evolution. Curr Anthropol 25: 483–495.

[2] Bramble DM, Lieberman DE (2004) Endurance running and the evolution of Homo. Nature 432: 345–352. 15549097 10.1038/nature03052

[3] Kuttruff JT, DeHart SG, O'Brien MJ (1998) 7500 years of prehistoric footwear from Arnold Research Cave, Missouri. Science 281: 72–75. 9651246 10.1126/science.281.5373.72

[4] Trinkaus E, Shang H (2008) Anatomical evidence for the antiquity of human footwear. J Archaeol Sci 35: 1928–1933.

[5] Bennett MR, Harris JWK, Richmond BG, Braun DR, Mbua E, et al (2009) Early hominin foot morphology based on 1.5-million-year-old footprints from Ileret, Kenya. Science 323: 1197–1201. 19251625 10.1126/science.1168132

[6] Richmond BG, Bennett MR, Harris JWK, Behrensmeyer AK, Braun DR, et al (2010) The anatomy of footprints from Koobi Fora, Kenya. Am J Phys Anthropol 141: 197.

[7] Roberts DL (2008) Last Interglacial Hominid and Associated Vertebrate Fossil Trackways in Coastal Eolianites, South Africa. Ichnos 15: 190–207.

[8] Richmond BG, Hatala KG, Harcourt-Smith WEH, Rossi V, Metallo A, et al (2011) Early modern human footprint assemblage from Engare Sero, Tanzania. PaleoAnthropology 2011: A31.

[9] Hatala KG, Richmond BG, Harcourt-Smith WEH, Rossi V, Metallo A, et al (2011) Early modern human footprints from Engare Sero, Tanzania. Am J Phys Anthropol S52: 158.

[10] Webb S, Cupper ML, Robins R (2006) Pleistocene human footprints from the Willandra Lakes, southeastern Australia. J Hum Evol 50: 405–413. 16343597 10.1016/j.jhevol.2005.10.002

[11] Hoffman P (1905) Conclusions drawn from a comparative study of the feet of barefooted and shoe-wearing peoples. Am J Orthop Surg 3: 105–137.

[12] Barnett C (1962) The normal orientation of the human hallux and the effect of footwear. J Anat 96: 489–494. 13969386 PMC1244092

[13] Ashizawa K, Kumakura C, Kusumoto A, Narasaki S (1997) Relative foot size and shape to general body size in Javanese, Filipinas, and Japanese with special reference to habitual footwear types. Ann Hum Biol 24: 117–129. 9074748 10.1080/03014469700004862

[14] D'Août K, Pataky TC, De Clercq D, Aerts P (2009) The effects of habitual footwear use: foot shape and function in native barefoot walkers. Footwear Sci 1: 81–94.

[15] Lieberman DE, Venkadesan M, Werbel WA, Daoud AI, D'Andrea S, et al (2010) Foot strike patterns and collision forces in habitually barefoot versus shod runners. Nature 463: 531–535. 20111000 10.1038/nature08723

[16] Daoud AI, Geissler GJ, Wang F, Saretsky J, Daoud YA, et al (2012) Foot strike and injury rates in endurance runners: A retrospective study. Med Sci Sports Exerc DOI: 10.1249/MSS.0b013e3182465115. 10.1249/MSS.0b013e3182465115 22217561

[17] Milner CE, Ferber R, Pollard CD, Hamill J, Davis IS (2006) Biomechanical factors associated with tibial stress fracture in female runners. Med Sci Sports Exerc 38: 323–328. 16531902 10.1249/01.mss.0000183477.75808.92

[18] Davis IS, Bowser B, Mullineaux D (2010) Do impacts cause running injuries? A prospective investigation. Annual Meeting of the American Society of Biomechanics

[19] Cunningham CB, Schilling N, Anders C, Carrier DR (2010) The influence of foot posture on the cost of transport in humans. J Exp Biol 213: 790–797. 20154195 10.1242/jeb.038984

[20] Perl D, Daoud AI, Lieberman DE (2012) Effects of footwear and strike type on running economy. Med Sci Sports Exerc 44: 1335–1343. 22217565 10.1249/MSS.0b013e318247989e

[21] Nigg BM, Bahlsen HA, Luethi SM, Stokes S (1987) The influence of running velocity and midsole hardness on external impact forces in heel-toe running. J Biomech 20: 951–959. 3693376 10.1016/0021-9290(87)90324-1

[22] Keller TS, Weisberger AM, Ray JL, Hasan SS, Shiavi RG, et al (1996) Relationship between vertical ground reaction force and speed during walking, slow jogging, and running. Clin Biomech 11: 253–259. 10.1016/0268-0033(95)00068-2 11415629

[23] Herzog W (1979) The influence of running speed and running surface on the load of the human body. Zurich: Federal Technical Institute.

[24] Liebenberg L (2006) Persistence hunting by modern hunter-gatherers. Curr Anthropol 47: 1017–1025.

[25] Pickering TR, Bunn HT (2007) The endurance running hypothesis and hunting and scavenging in savanna-woodlands. J Hum Evol 53: 434–438. 17720224 10.1016/j.jhevol.2007.01.012

[26] Lieberman DE, Bramble DM, Raichlen DA, Shea JJ (2007) The evolution of endurance running and the tyranny of ethnography: a reply to Pickering and Bunn (2007). J Hum Evol 53: 439–442. 17767947 10.1016/j.jhevol.2007.07.002

[27] Steudel-Numbers KL, Wall-Scheffler CM (2009) Optimal running speed and the evolution of hominin hunting strategies. J Hum Evol 56: 355–360. 19297009 10.1016/j.jhevol.2008.11.002

[28] Alexander RM, Jayes AS (1983) A dynamic similarity hypothesis for the gaits of quadrupedal mammals. J Zool 201: 135–152.

